# The Need to Focus on Therapy Instead of Associations

**DOI:** 10.3389/fcimb.2019.00327

**Published:** 2019-09-12

**Authors:** Gregor Reid

**Affiliations:** ^1^Canadian R&D Centre for Human Microbiome and Probiotics, Lawson Health Research Institute, London, ON, Canada; ^2^Departments of Microbiology and Immunology, and Surgery, Western University, London, ON, Canada

**Keywords:** vaginal dysbiosis, microbiota, *Lactobacillus*, probiotics, prebiotics

## Abstract

Molecular analyses of the vaginal microbiota have uncovered a vast array of organisms in this niche, but not so far changed what has been known for a long time: lactobacilli are dominant in health, and the diagnosis and treatment of symptomatic bacterial vaginosis is sub-optimal, and has not changed for over 40 years. While the lowering cost of DNA sequencing has attracted more researchers to the field, and bioinformatics, and statistical tools have made it possible to produce large datasets, it is functional and actionable studies that are more urgently needed, not more microbial abundance, and health or disease-associative data. The triggers of dysbiosis remain to be identified, but ultimately treatment will require disrupting biofilms of primarily anaerobic bacteria and replacing them with the host's own lactobacilli, or health-promoting organisms. The options of using probiotic strains to displace the biofilms and for prebiotics to encourage resurgence of the indigenous lactobacilli hold great promise, but more researchers need to develop, and test these concepts in humans. The enormity of the problem of vaginal dysbiosis cannot be understated. It should not take another 40 years to offer better management options.

With the advent of less expensive microbial sequencing methodologies, the volume of data being produced on which microbes inhabit the vagina, bladder, and other human body sites increases exponentially. But are these leading us closer to improved treatment or are they just delaying this long-awaited step?

A recent study of vaginal samples from 45 pregnant women who delivered preterm and 90 who delivered at term (Fettweis et al., 2019) concluded an association between *Sneathia amni* and a few other bacteria along with depleted *Lactobacillus crispatus*. As intensive as the study was in terms of human-power and techniques used, the impressive processing of over 200,000 samples from women and neonates, and the enormity of the expense, the end result is an unproven cause and effect with no path to practical action.

If indeed anaerobic bacteria such as *Sneathia*, are responsible for preterm birth, why have studies using antibiotics failed to prevent this outcome? If adequate clinical trials have not fully tested this theory, what agents are available to specifically target, and eradicate anaerobes such as *Sneathia*? The answer is none. Indeed, the pharmaceutical armamentarium for treating vaginal dysbiosis or symptomatic bacterial vaginosis (BV) has been inadequate for 40 or more years. There lies the real problem.

For all the vast number of characterizations of the vaginal microbiota, few surprises have emerged. Indeed, culture analysis from decades ago have informed us that lactobacilli are dominant in health in most women, and depleted and replaced by anaerobes or other pathogens in “disease” (Pfau and Sacks, 1977; Hill et al., 1984).

In a recent report, the “disease” of BV was questioned as being a catch-all for several conditions, one of which may be infection (Reid, 2018). This therefore counters the very topic of this collection of papers and states that BV is not in fact a true polymicrobial infection. Rather, one component of the term includes an infectious state, but many other conditions that are not infectious or symptomatic are still referred to as BV. A call to re-define BV was made. One sub-group certainly fits as being an infection requiring treatment, but this relies on appropriate diagnosis which is currently far from effective.

## Failure of Diagnostic Techniques

Indeed, credit to Fettweis et al. for not using the Nugent score (Gram stain assessment of bacteria on vaginal cells) or Amsel criteria (discharge, microscopic assessment of cells, whiff test and pH) to diagnose BV as they depend on the experience of the reader and even then cannot distinguish bacterial types effectively. Instead, they use the term “dysbiosis” as we proposed 4 years ago (McMillan et al., 2015). The result was that changes in bacterial diversity and taxa abundance were reported.

The net effect of this for clinical practice, however, is that assessment of patients would require analysis of their vaginal swab using a system that presently is not rapid, easily available, or affordable. This again speaks to the actionable outcomes of such studies. In the not so distant future, rapid “diagnostic” tools will be developed to identify the microbes present on vaginal swabs, and more importantly the metabolic profile. The identification of molecules like gamma hydroxybutyrate or amines (McMillan et al., 2015) could well-prove more useful for clinical assessment. But as noted above, given the lack of targeted therapies, this would still be insufficient to optimally manage the patient.

## Too Much Emphasis on Community State Types and Not Enough on Restoration and Maintenance of a Healthy Vagina

One of the outcomes of microbiota studies has been an effort to try and create community state types (Ravel et al., 2011). The original five types are now up to 13 and could increase further once more samples are processed. The problem is these are based on statistical associations rather than a direct means to identify patients who require intervention. Given the therapeutic options available, essentially metronidazole and clindamycin, and the lack of targeted drugs to only kill *Sneathia, Atopobium, Prevotella*, or whatever other etiological agent falls into one of the state types, then the groupings are not informative.

Rather, the issue that has existed for decades is why do these organisms primarily anaerobes, propagate and form biofilms (Swidsinski et al., 2013), and how can this be prevented or better treated so that ecological equilibrium is restored? The trigger for a *L. crispatus*-dominated vaginal microbiota to be displaced and replaced by *Gardnerella* and these other anaerobes is completely unknown. Douching, sexual intercourse, spermicides, stress, urinary tract infections and numerous other “causes” have been proposed (Hickey et al., 2012) but again these are associations and not molecularly proven triggers. Research is needed to answer this important question.

The emphasis on *L. crispatus* as being “protective” because it is most associated with a healthy vagina and therefore the optimal choice as a probiotic intervention, is not particularly based on properties of the species. Indeed, selection of candidate probiotic strains seems to be their ability to adhere to vaginal cells and produce lactic acid and hydrogen peroxide (Hemmerling et al., 2009). Genomic and metabolomic studies have shown that there are significant differences between *L. crispatus* strains (van der Veer et al., 2019; Watson et al., unpublished), so some selection criterion is needed before expecting a strain to be effective. If the species is so protective, why is it apparently readily displaced by anaerobes? Arguably strains with anti-pathogen properties capable of disrupting BV biofilms would be a better choice, irrespective of their species (McMillan et al., 2011). This was the rationale for developing *Lactobacillus rhamnosus* GR-1 and *Lactobacillus reuteri* RC-14 for urogenital application with positive clinical outcomes reported (Reid, 2017). At the very least, it was an attempt at intervention, prevention and even treatment based upon strain properties with a rationale (Reid, 2017; Petrova et al., 2018).

## The Options Are Not Futuristic

Some researchers are reluctant to go to human studies until the ultimate “magic bullet” has been found. I would argue that this discovery will never happen, but in the meantime hundreds of millions of women with continue to suffer from malodour, discharge, and the other signs, and symptoms that occur with dysbiosis.

This is not to suggest the testing of products with no plausible documentation as to why they would work. Safety and ethics are paramount.

But, if after all this time we know the problem (malodour, discharge, discomfort) that takes women to seek medical assistance, and we know the causative agents are anaerobic bacteria, plus we know that “normal” is a lactobacilli-dominated vagina, why have more therapies not been developed?

Part of the answer lies within the regulatory system that in Europe and the United States takes the outdated view that only drugs can treat, prevent, cure or mitigate disease. Thus, even if a probiotic food or supplement was to show disease efficacy, it would not be allowed to make that claim until it had been registered and processed as a drug. This almost always requires animal sacrificies, and with no animal model for vaginal dysbiosis, this becomes a problem for developing therapies. The very nature of regulatory agencies means that the assessment tends to fall under biological agents as if lactobacilli generally regarded as safe were somehow equivalent to *Salmonella*. The end result is a process of drug approval that is enormously expensive and time-consuming. As the profit margins of food and supplement companies is invariably significantly smaller than for drug companies, this acts a deterrent.

Clearly pharmaceutical companies have so far avoided developing new vaginal therapies, in part because no suitable classes of antibiotics have emerged, and because there is a perception that probiotic strains and products cannot be patent protected to allow market exclusivity. The latter is of course incorrect and companies like BioGaia have been operating for decades based upon intellectual property for *L. reuteri* strains. In terms of return on investment, the enormity of the clinical problem and lack of suitable treatments should be sufficient for companies, and the profitability of clotrimazole, and miconazole nitrate over-the-counter treatments for vulvovaginal candidiasis serves as a precedent.

Some probiotic strains are available as food or supplements with the intent of either reaching the vagina via ascension from the rectum or reducing pathogen re-seeding, or through a systemic immune, or other mechanism. Unfortunately, a consequence of current regulations is that non-drug products, even those highly documented in human studies, cannot be differentiated on the label from those with no evidence of benefit. This makes it difficult for women and physicians to know the studies that support treatment, prevention or mitigation of disease. As physicians are trained to prescribe drugs and there is oversight in manufacturing and an expectation of safety and effectiveness with them, they may dismiss probiotics *per se* rather than considering some for their potential value to the patient. A clinical guide has been prepared by a group of experts who reviewed only products available in Canada (and another guide for the U.S.) that have been tested in humans. The list gives the level of evidence and citations are provided to help consumers and healthcare providers select documented probiotics (Clinical Guide to Probiotic Products Available in Canada, 2019).

In Canada at least, three ovule products for vaginal insertion have been approved. These comprise *L. acidophilus* A-212, with *L. rhamnosus* A-119, and *S. thermophilus* A-336; *L. rhamnosus* Lcr35; and *L. rhamnosus* PBO1, *L. gasseri* EN-153471 (EB01). The clinical evidence in support of their approval by Health Canada for use in BV is not readily available on sources such as PubMed, so it is unclear how the review panel that prepares the Clinical Guide had sufficient data to include them in the list. The exception is a study of Lcr35 administered daily for 7 days as a suppository following clindamycin treatment of BV (Petricevic and Witt, 2008). The outcome was only assessed at 4 weeks with 69 of 83 women (83%) having at least a five-grade reduction of the Nugent score compared to 31 of the 88 women (35%) in the control group. The selection of the strain appeared to be primarily based on its intestinal probiotic activity plus the possession of a bacteriocin against *Gardnerella vaginalis* (Turovskiy et al., 2009). It is unusual for a Gram-positive bacterium to produce a bacteriocin against a Gram-negative species, so it is unfortunate that the killing of *G. vaginalis* following Lcr35 application was not verified in the human study. Such mechanistic evidence and a longer follow-up would be more convincing in supporting the use of this product to treat BV without antibiotics, or to prove it allows recovery of healthy microbiota to equilibrium after drug therapy.

Only one strain combination, *L. rhamnosus* GR-1, and *L. reuteri* RC-14, has been tested to actually cure symptomatic BV without the use of antibiotics. In a randomized trial, 40 women diagnosed with BV by discharge, fishy odor, sialidase positive test, and Nugent Gram stain scoring, were randomized to receive the probiotic in capsules daily for 5 days, or 0.75% metronidazole gel, applied vaginally twice a day (in the morning and evening) (Anukam et al., 2006). The 90% cure rate with probiotics was superior to the antibiotic. If this result was to be confirmed in a second, larger study, it would provide strong evidence that probiotics can be an alternative to antibiotics.

In terms of issues of safety, these of course cannot be overlooked no matter the regulatory path. The administration of live organisms to humans is never completely without risk, and the ease of eradication of lactobacilli, and bifidobacteria by antibiotics reduces the risk of serious complications. Some risk can be mitigated by carefully testing the strains for virulence factors or drug resistance before their application to at-risk patients. Nevertheless, there are cases of both these genera being associated with infection (Omar et al., 2019; Pruccoli et al., 2019). Thus, recommendations as to how to deal with higher risk recipients of probiotic strains should be considered (Sanders et al., 2016). The potential to use prebiotic compounds (a substrate that is selectively utilized by host microorganisms conferring a health benefit) (Gibson et al., 2017) has been explored to a much lesser extent. The challenge is the degree to which lactobacilli have been depleted in dysbiosis. If there are sufficient present to be resurrected by prebiotics such as lactulose stimulating their growth (Collins et al., 2018), then this could potentially be a useful therapeutic approach. It would likely not be considered a drug unless disease claims were made, but it is also not considered a food since it is being administered to the vagina and the European Food Safety Authority have stated that the vagina does not require nutrition (Reid, 2011).

## Ultimately

Microbiome studies have informed us of the organisms that are present in the vagina, and to a lesser degree what they are doing. The same is true for multiple studies of the gut microbiota where the failure to translate to clinical interventions is all too apparent (Brussow, 2019). Metabolomic studies may be more informative than microbiota profiles to “diagnose” an aberration that requires treatment or to show that a treatment has succeeded. But the complexity of the reproductive tract with the menstrual cycle, hormonal changes, exposure to sexual partners and the environment, presents many challenges that will require a variety of options for health restoration and maintenance over the lifespan.

This does not mean a single *Lactobacillus* or a cocktail of strains or a mixture with prebiotics, cannot make a significant dent in the symptomatic dysbiotic conditions. There is sufficient evidence to already indicate this is possible before, during and after pregnancy (Reid et al., 2016).

Going forward, the onus is on the research community to select candidate strains/products, pilot test them in humans, and understand how they succeed or why they fail. The methods of selection have advanced with metabolomic and transcriptomic techniques (Macklaim et al., 2013; McMillan et al., 2015) which can differentiate lactobacilli strains more suited to the vagina (Watson et al. unpublished) and may one day benefit from tissues on a chip technology (Maurer et al., 2019). Funding from industry and government agencies should make this a priority given the extent of the problem and the failure to adequately manage it for so long. If only to make an impact in quality of life and to reduce episodes of dysbiosis and any symptoms and signs associated with it, then current and future products will have made an important contribution to women's health.

## Author Contributions

The author confirms being the sole contributor of this work and has approved it for publication.

### Conflict of Interest Statement

No personal, professional or financial assistance was provided to prepare this manuscript. The author developed probiotic strains GR-1 and RC-14 but has had no financial stake in them for over 11 years. He is currently Chief Scientific Officer of Seed, a company producing probiotic products that are not mentioned in this article. Over the past 3 years, he has consulted on probiotics with Acerus Pharmaceuticals, Altmann, Chr. Hansen, Danone, KGK Science, Kimberly-Clark, Metagenics, and Seed.
